# Simplifying checkpoint inhibitor delivery through *in vivo* generation of synthetic DNA-encoded monoclonal antibodies (DMAbs)

**DOI:** 10.18632/oncotarget.26535

**Published:** 2019-01-01

**Authors:** Alfredo Perales-Puchalt, Elizabeth K. Duperret, Kar Muthumani, David B. Weiner

**Affiliations:** ^1^ Vaccine and Immunotherapy Center, The Wistar Institute, Philadelphia, PA, USA

**Keywords:** immunotherapy, cancer, immune checkpoints, DMAb, monoclonal antibody

## Abstract

Checkpoint inhibitors (CPI) have revolutionized the treatment of many solid tumors. However, difficulties in production, stability, the requirement of frequent high doses for antibody administration and long intravenous administration are recurring issues. Synthetically designed DNA-encoded monoclonal antibodies (DMAbs) are a novel delivery method for antibody therapy which could potentially address many of these issues, simplifying design and implementation of MAb-based therapies. DMAbs delivered through plasmid DNA injection and electroporation have been used in preclinical models for the treatment or prophylaxis of infectious diseases, cancer and cardiovascular disease. Our group has recently reported that immune checkpoint blockers can be optimized and delivered *in vivo* advancing further DMAb technology by optimization, expression and *in vivo* functional characterization of anti-CTLA4 antibodies. Here we report optimization, expression and binding of DMAbs based on anti-PD1 CPI and discuss the potential of DMAbs in checkpoint immunotherapy.

Checkpoint inhibitors (CPI) have revolutionized the treatment of many solid tumors. These therapies have generated an increase in the percentage of cancer long-term survivors by ‘releasing the brakes’ of the immune system to potentiate its antitumor effect in those patients who respond [1]. However, difficulties in production, stability, the requirement of frequent high doses for antibody administration and long IV administration requirements are recurring issues. These can limit the number of patients treated with this game changing tool and slow the study of CPI combination approaches [2]. Collectively, such issues contribute to high cost of these products, for example, in the United States, checkpoint inhibitor therapy costs $100,000-$200,000 per year, and combination therapies which can demonstrate additional synergistic effects, would add complexity and cost to this approach [3].

Synthetically designed DNA-encoded monoclonal antibodies (DMAbs) are a novel delivery method for antibody therapy which could potentially address many of these issues, as well as open up novel approaches for design and implementation of MAb based therapies. DMAbs represent a simple, scalable development system, with the additional benefit of higher stability and simpler storage than protein biologicals. DMAbs are administered locally by intramuscular injection and allow simple coformulation, permitting the generation of CPI with a unique profile of expression and potency, suggesting they could be considered as a new tool for study in this important therapeutic arena.

DMAbs delivered through plasmid DNA injection and electroporation (EP) have been used in pre-clinical models for the treatment or prophylaxis of infectious diseases [4, 5], cancer [4, 6, 7] and cardiovascular disease [8]. These preclinical studies have demonstrated significant levels of expression of circulating DMAb launched antibodies in mouse serum that are stable for several months [6]. This suggests that a single DNA injection could provide coverage for long periods, decreasing the need for multiple visits for drug administration, and as injection is rapid, decreasing the time required for each patients’ dosing. Thousands of persons have been treated using DNA plasmids and DNA delivery technology in the clinic for vaccine and immunotherapy studies establishing an important favorable safety profile for this approach [9].

In support, Duperret et al., recently reported that immune checkpoint blockers can be optimized and delivered *in vivo* advancing further DMAb technology [6]. In the study, Duperret and collaborators optimized, expressed and characterized 9D9 a mouse CPI targeting CTLA4. They studied the effects of the 9D9 DMAb directly produced in the animals launched from Cellectra EP® delivered plasmids for expression and duration as well as in cancer therapy in models responsive to mouse CPI therapy. The anti-CTLA4 DMAbs maintained binding and exhibited a profound antitumor effect both shrinking tumors as well as significantly increasing the life span of the tumor challenged animals. As a follow-up transition study, synthetic DNA sequences were developed to launch DMAb versions engineered as antibody replicas of human anti-CTLA4 therapeutics for either ipilimumab [10] or tremelimumab [11]. These studies demonstrated that a single local intramuscular injection of the DMAb encoding an ipilimumab-like molecule achieved serum levels greater than those associated with tumor impact in the clinic. The expression levels persisted at 20ug/ml levels for several months post a single injection. In the clinic, the recommended regimen for ipilimumab delivery is a 3mg/kg dose administered intravenously over 90 minutes every 3 weeks for a total of 4 doses, and the mean ipilimumab serum concentration achieved at steady state with this dose is 21.8ug/mL (Bristol-Myers Squibb investigational brochure). This maintenance dose is within the range observed by the encoded DMAb-Ipi in mice. Similar results were observed with a Tremelimumab-like DMAb. A single delivery of anti-CTLA4 DMAb resulted in potent CTLA-4 blocking activity when the mouse serum was screened on human target cells, very similar to the bioproduced molecules, except delivered in a simpler regime with *in vivo* expression for several months. These first studies in mice, while encouraging, require additional examination in other models, including additional optimization in other species to further understand the implications with an eye towards subsequent clinical evaluation. Furthermore, studies in combinations and with additional genetic modifications for functional enhancement by the Fc portion of the Ig heavy chain can be much more easily developed and studied as DMAbs than as protein biologicals to advance new CPI-like forms with improved functions resulting in improved tumor impact advancing clinical benefit. Under this concept the development of additional CPI targets as DMAbs appears important.

The PD-1-PD-L1 signaling pathway was first discovered in 1992 and monoclonal antibodies targeting this suppressive pathway were moved towards clinical testing in 2006. These MAb have made a major positive impact in the clinic resulting in FDA approvals for treating a variety of different tumor types due to exciting clinical benefit for treatment of diverse cancer types including melanoma, non-small cell lung carcinoma, renal cell carcinoma, high microsatellite instability cancers, Hodgkin’s disease, hepatocellular carcinoma, urothelial carcinoma, bladder carcinoma, Merkel cell carcinoma and gastroesophageal junction [1] with additional targets soon to come. CTLA-4 blockade and PD-1 blockade have demonstrated synergy and non-redundancy in pre-clinical mouse models and in patients. Due to the non-redundancy in these immune checkpoint pathways, the combination therapy of nivolumab (anti-PD1) and ipilimumab (anti-CTLA4) resulted in improved efficacy, including overall survival and progression free survival compared to monotherapy for the treatment of advanced melanoma, resulting in the FDA approval for this combination therapy in 2015.

Accordingly, the development of forms of DMAbs encoding anti-PD-1 like antibodies are important. Engineered DMAb forms mimicking highly impactful anti-PD-1 monoclonal antibodies were studied in an effort to optimize direct DMAb *in vivo* expression while retaining their required binding ability and functionality. These DMAbs expressed for several months and exhibited the specificity and cell binding of the parental antibodies. The recent development of DMAbs encoding anti-CTLA4 [6] and for anti-PD-1 (Figure 1) provide new tools for research aimed towards advancing of a new generation of CPI providing multiple benefits such as simplifying the patient regime for checkpoint blockade therapy, converting 90-minute intravenous infusions administered every three weeks into a single local injection requiring less frequent infusions, and possibly resulting in more consistency and through additional engineering ultimately providing improved benefits for the patient. DMAbs can also conceptually simplify combination therapies, allowing administration of anti-CTLA4 and anti-PD-1 combinations with a single injection or providing simplified immune combinations for study such as cancer vaccines [5, 12] or DMAbs targeting other tumor targets [4]. The ability to combine the power of genomics and advanced antibody engineering for simple and rapid *in vivo* production is likely to provide unique research and ultimately clinical benefit in the cancer arena.

**Figure 1:**
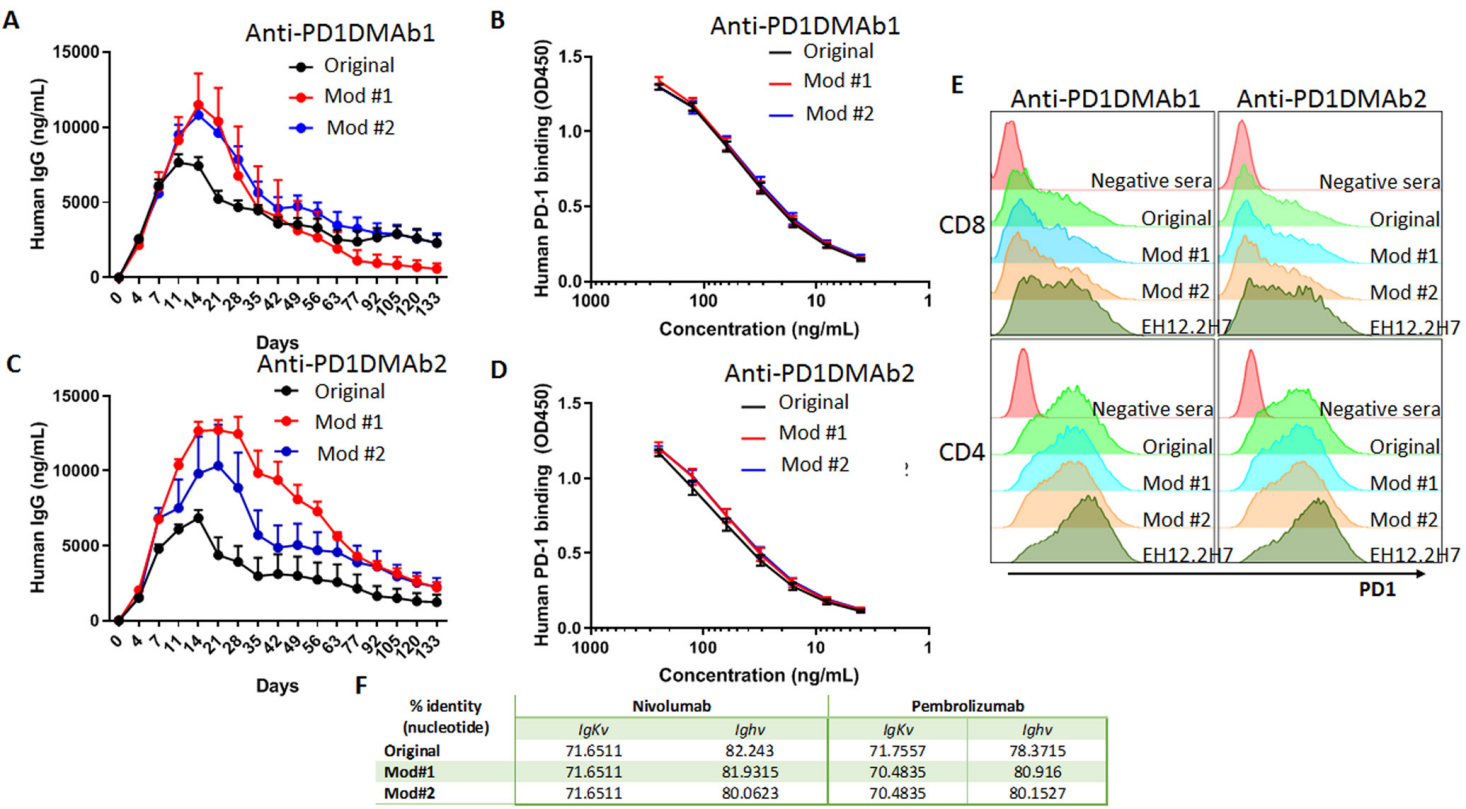
Expression and binding of human anti-human PD-1 DMAbs **A.** & **C.** Serum concentration of human IgG: anti-PD1DMAb-1 (based on Nivolumab) or anti-PD1DMAb-2 (based on Pembrolizumab) and its optimized sequences over time in Balb/c mice injected with 100ug of the indicated DMAb by IM-EP. (*n* = 5 mice per group). **B.** & **D.** Binding of anti-PD1DMAb-1 and 2 and their modifications purified from mouse serum to human PD-1 protein by ELISA. (*n* = 2 mice per group). Mean ±SEM. **E.** Binding of sera from mice injected with anti-PD1DMAb-1 or anti-PD1DMAb-2 original and modifications, untreated mice (negative sera) and anti-PD-1 antibody EH12.2H7 (positive control) by flow cytometry to CD8 and CD4 T cells after stimulation with anti-CD3/anti-CD28 beads for five days. **F.** Percentage of identity between the nucleotide sequences of Nivolumab and Pembrolizumab and anti-PD1DMAb-1 and 2 and their modifications. Mod: modification, IgKv: variable fragment of the kappa light chain, IgHv: variable fragment of the heavy chain.

In summary, DMAbs are a new tool that conceptually simplifies antibody therapies and their combinations, including checkpoint blockade, which if successful would likely expand their applications bringing CPI therapies to a much larger patient population. Further translational study of the DMAb platform for impacting cancer is worthy of consideration.
